# Treatment of Alcoholism by Strychnine Nitrate

**Published:** 1896-06

**Authors:** 


### Treatment of Alcoholism by Strychnine Nitrate.

Dr. Breed thinks that we have in nitrate of strychnine a remedy for alcoholism that is safer, and at the same time gives as good results as the gold-cure, Keeley cure, silver-ash cure, etc. It is a treatment that is no secret, and can be used by the profession generally. This drug removes the desire for alcoholic stimulation for, as yet, an undetermined length of time without the least effort on the part of the patient; though, of course, it cannot oblige a man to abstain if he is determined to continue the use of alcohol. Nitrate of strychnine removes the distress and gnawing at the epigastrium so common upon the withdrawal of alcohol; it tones up the nervous system and allays tremulousness and uncertainty of voluntary motions; it restores the appetite and general physical vigor and is, incidentally, a good heart tonic. These latter effects restore hope and self-confidence in the patient, and these in turn aid much in his recovery.—*Medical News.*

In compliance with the excise law, the sale of brandy drops, or other confections containing intoxicating liquors, has been ordered discontinued in New York by the police.

